# Evidence of BVDV in Pigs from North Eastern Part of India- Genetic Profiling and Characterisation

**DOI:** 10.2174/1874357901812010110

**Published:** 2018-08-31

**Authors:** Amit Kr Chakraborty, Priyanka Mukherjee, Amarjit Karam, Samir Das, Luit Barkalita, Kekungo Puro, Rajkumari Sanjukta, Sandeep Ghatak, Ingudam Sakuntala, Ram Gopal Laha, Prabodh Borah, S.V. Ngachan, Indu Sharma, Arnab Sen

**Affiliations:** 1 Division of Animal Health, ICAR Research Complex for NEH, Barapani - 793103, India; 2 Department of Microbiology, Assam University, Silchar - 788011, India; 3 Department of Biotechnology, C.V.Sc, AAU, Khanapara, Assam, India

**Keywords:** Bovine viral diarrhea virus, Porcine, North east, Sequence analysis, Phylogenetic analysis, India

## Abstract

**Introduction:**

The work has been attempted to detect and genetically characterise the nature of Bovine Viral Diarrhea Virus (BVDV) isolates from the porcine population of the north east.

**Methods and Material:**

The samples have been collected over a two year period and are from areas where there is a mixed and integrated rearing of livestock in close proximity. The isolates were identified, cloned and sequenced using BVD specific genomic primers for two important domains viz., E-2 and 5’ UTR.

**Results:**

Porcine BVD Sequences were analysed phylogenetically. Divergence in 3 sequences is noted in the 5’ UTR region that are forming a clear outlier group while E-2 sequences are coming close to BVDV group but forming a separate cluster.

## 1 INTRODUCTION

Interspecies viral spill-over is responsible for most of the emerging infections. Emergence of new disease or new viral pathogens is often a result of species jumping or cross species transmission of viral pathogens.Successful cross species transmission of a virus is a relatively rare phenomenon as it must overcome the ecological and evolutionary barriers in the process to establish itself into a new host [1]. This phenomenon can play a major role in Virus evolution particularly in case of retroviruses which is related to their host cell morphology suggesting their long term Co divergence as per their phylogenetic tree [2], while in case of Flaviviruses host switch over is more frequent among other RNA viruses as many of them transmitted by arthropod vectors and cause a short term infection [3].

Bovine Viral Diarrhoea Virus (BVDV), a member of Flaviviridae family under genus Pestivirus, one of themost clinically and economically important ruminant pathogen. It was confirmed that BVDV and CSFV (another Pestivirus cause havoc mortality in pigs worldwide) shares cross reactive antigens [4]. Fernetius *et al*. in 1973 sucessfully isolated BVDV from pigs in Australia. In Asia BVDV in pigs was first reported in 1996 [5] in China. In India however the scenario is quite unknown. Pestivirus like BVDV, CSFV and BDV are antigenically close to each other and cross reaction or species spill over are often found [6].

Integrated farming systems are the mainstay of north eastern agriculture sector. Co-habitation of small livestock holds is quite common place. It is very common to see a herd of cattle co-existing with porcine in the near vicinity. This practice is unique to the region and could well serve in bridging niche-gaps leading to cross species transmission. We conducted this study here to observe if any such scenario is happening.

BVDV can be found in cattle, sheep, swine, goats, and other wild animals [7]. BVDV in pigs can create smiler symptoms like classical swine fever and thus cause difficulty while differentiating from each other [8]. But mainly BVDV in pigs shows no significant signs thus it has the advantage to spread without getting noticed [9], [10].

## 2 MATERIAL AND METHODS

### 2.1 Origin of Samples

Pig serum samples (n=206) were randomly collected from villages or from organised farms of Meghalaya (n=82), Assam (n=31), Mizoram (n=44), Nagaland (n=6), Manipur (n=12) and Arunachal Pradesh (n=31) for a period of two years (2013-15). Each sample was aseptically collected from ear veins in a vacutainer using sterile precaution and was transferred in -200C gel packs to the laboratory within 24 to 48 hours. The samples were collected over a two year period (2013 – 2015). These samples were collected from areas that had integrated farming operations and a mixed livestock population comprising of bovines, porcines and poultry. In the north eastern region small holder farms with livestock being reared in close proximity is quite common. Hence the samples were obtained from these farms or zones of integrated farming practice.

The protocols followed in the study were OIE approved as per genomic detections of BVD in are concerned. (OIE Manual, 2015). Standardised primers and validated methods of detection of BVD in porcines as reported by earlier workers were followed.

### 2.2 Isolation of RNA and Detection by RT PCR

Viral RNA were extracted from serum samples using Qia Amp Viral RNA mini kit (Qiagen, Germany) following manufacturer’s instructions and subjected to the synthesis of cDNA using Revert Aid First strand cDNA synthesis kit (Thermoscientific, USA) with random hexamer primers. These cDNA’s were then checked with a set of BVDV specific nested primers namely Pan Pesti and then Pan BVDV and Pan CSFV (Table **1**) to detect the presence of BVDV and CSFV respectively by amplifying the 5’UTR region of both the viruses, as both the specific primers are specific for a partial region of 5’UTR. The initial identity was checked again using PnPesti, subsequently PnBVDV and PnCSF were used to ascertain whether the nucleic acids were from BVD and/or CSFV. The cyclic conditions which are same for the three sets of primers are primary denaturation at 950C for 3 minutes followed by 40 cycles at 950C for 30 second, 550C for 30 seconds, 720C for 30seconds and final extension at 720C for 5 minutes. DreamTaq green from Thermoscientific, USA was used for PCR. The products were then observed in a 2% agarose gel. After detection partial E2 region of BVDV was amplified from PCR positive samples for further phylogenetic study.

### 2.3 Detection and Quantification of BVDV by RT qPCR

The positive samples from conventional PCR were subjected for absolute quantification using Anigen BVDV Real-Time Detection Kit from Bionote, Korea (cat no. PD65 050N) in a standard curve method following manufacturer’s instruction. qPCR was performed using Step One Plus Real Time PCR machine from Applied Biosystems, USA. The control positive BVDV RNA was supplied by the company with the kit. The experiment was cross checked using 10 RNA samples which were negative for BVDV and positive for CSFV as negative controls to determine kit’s specificity and confidence level. The negative controls were confirmed by conventional PCR using BVDV and CSFV 5’UTR detection primers listed in Table **1**. The Taqman primer probe was directed against specific genomic regions of BVDV albeit the exact genetic sequences of the primer probe sets were not disclosed by the manufacturer.

### 2.4 Cloning, Nucleotide Sequencing and Phylogenetic Analysis

The positive RNA samples obtained in Taqman real time PCR kit were then further amplified with Pan Pesti forward and PnBVDV reverse primer in a hemi nested manner. A 232bp PCR product (5’NTR) and a 606bp PCR product (E2) then purified with TaKaRa PCR purification kit and purified fragment then ligated with pTZ57R/T (plasmid) vector (Thermoscientific, USA) and then DH5α competent cells (prepared with CaCl2treatment) were transformed with the plasmid containing insert fragment. Transformed colonies were isolated by blue white screening and plasmid were extracted with plasmid mini kit (Thermoscientific, USA) and then sequencing was done with ABI Big Dye Terminator v3.1 cycle sequencing kit (Applied Biosystems, USA) in ABI 3500xl genetic analyser. The sequences were assembled using Seqman software (DNASTAR Inc., Madison, USA). Additional pestiviral sequences were retrieved from NCBI GenBank and used in subsequent analysis. A total of eight E-2 partial gene sequences and eight 5’ UTR sequences were submitted to the Gen Bank during March. 2017 and provisional accession numbers were obtained. (KY852361-69/KY836194-200). These sequences were used for building the phylogenetic tree and derived protein data.

## 3 RESULTS

### 3.1 Conventional PCR

**Fig. (1):**
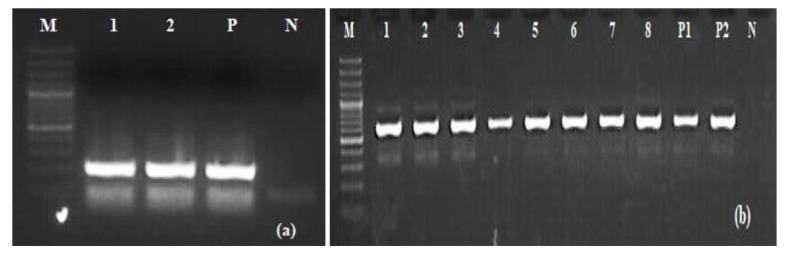
Agarose gel electrophoresis photo of BVDV specific gene amplification in pig samples. (**a**) Lane 1 and lane 2 are samples MzS1 (Mizoram) and ML1920 (Meghalaya). P is positive BVDV RNA sample. In M lane 100 bp plus ladder (Thermoscientific, USA) was used. (**b**) E2 partial 606bp fragment was amplified from pig serum. Lane 1 to lane 8 are samples from porcine origin and lane P1 and lane P2 are from bovine origin BVDV positive samples for verification of PCR. Lane N is non template control used in both (**a**) and (**b**).

Out of 206 porcine serum samples 10 (Meghalaya (n=4), Mizoram (n=6)) samples were showing presence of BVDV positive bands that consistently amplified with PnBVDV primers that were indicative of amplification of 5’UTR. However 8 among those 10 positive samples were also showing BVDV 606bp E2 specific bands (Fig. **1a**, **b**).

### 3.2 Real Time PCR

**Fig. (2):**
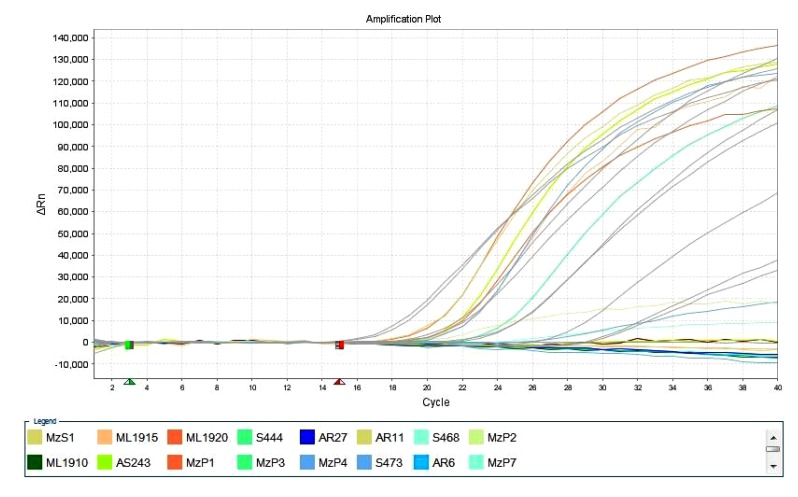
Amplification plot of Taqman RT qPCR of BVDV detection kit from.

These 10 samples further were confirmed as positive in Real Time PCR with Taqman BVDV detection kit. These 10 BVDV positive samples were cross checked along with 10 known negative controls for testing the specificity of the kit. Those 10known negative controls were negative in the test (Fig. **2**).

### 3.3 Phylogenetic analysis

**Fig. (3):**
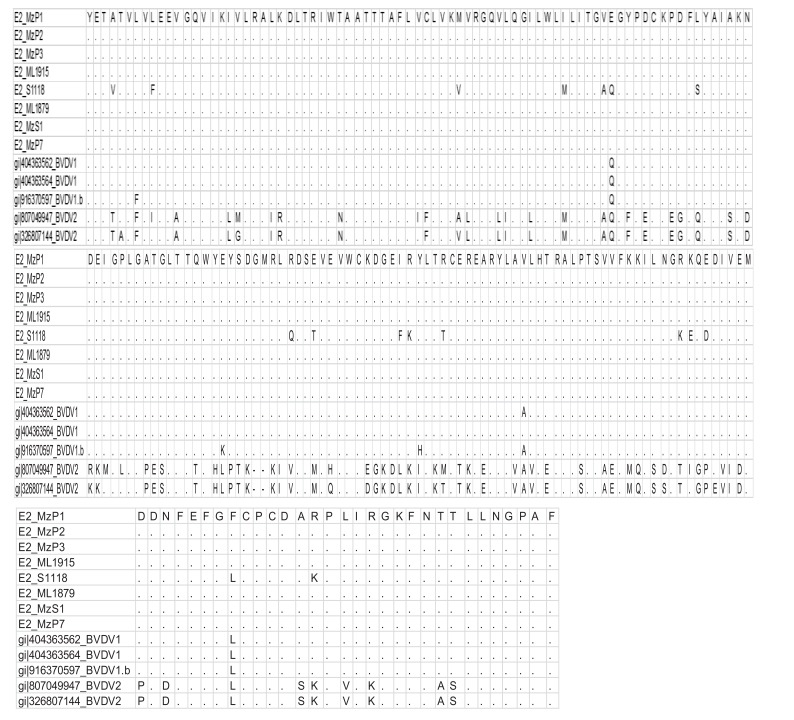
The representation of the alignment of protein sequence derived from the partial E2 sequences of BVDV from porcine isolates with the online available sequences.

Sequences were aligned in Molecular Evolutionary Genetics Analysis version 7.0 software [13] using clustal W method and tree were constructed using maximum parsimony [14]. Partial E2 sequences were converted to protein sequence using online translation tools and they were further aligned to observe if there was any difference in protein levels due to species adaptation (Fig. **3**).

**Fig. (4):**
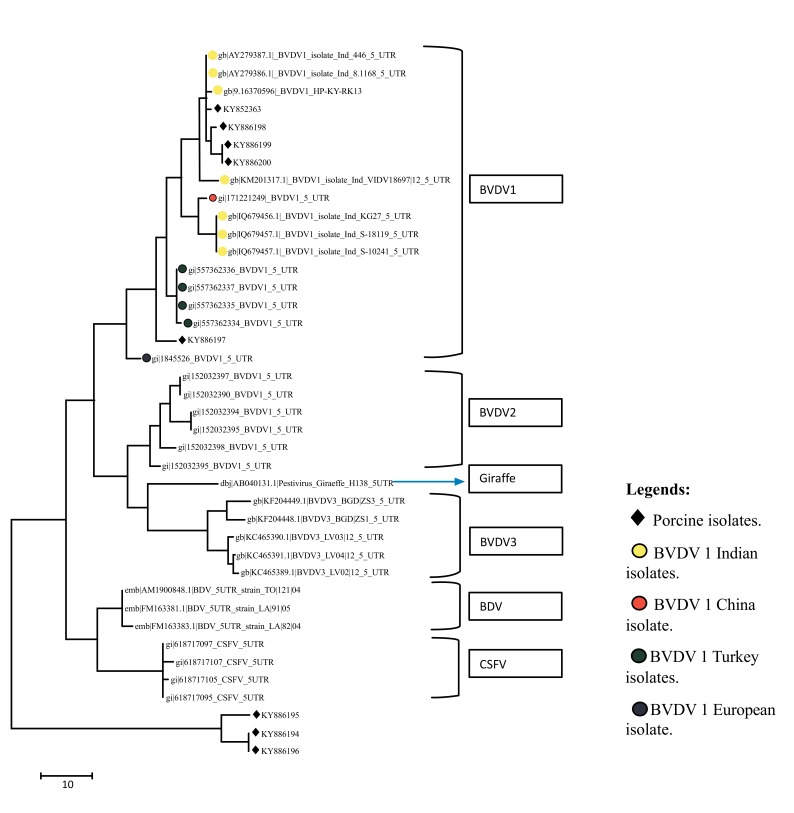
Phylogenetic diversity of BVDV based on 5’UTR sequences. The tree constructed using maximum parsimony method in MEGA ver.7.

**Fig. (5):**
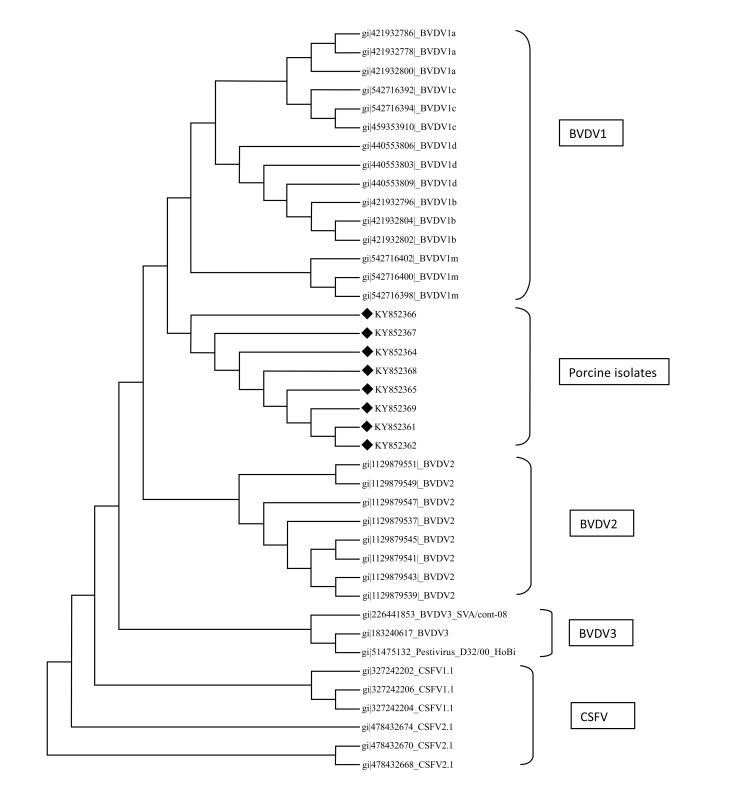
Phylogenetic diversity of BVDV based on 5’UTR sequences. The tree constructed using maximum parsimony method in MEGA ver.7.

A total of eight sequences for E-2 and eight sequences for 5’UTR were aligned using Clustal W method and phylogenetic trees were constructed using maximum parsimony statistical method. In the E-2 tree (Fig. **5**) it was seen that all the eight porcine BVD sequences were tightly clustered and were located close to BVDV 1 group of cattle. A comparison was done with reference isolates of BVD1, 2 and 3 (Cattle), BVDV, CSFV and Giraffe pestivirus (Fig. **4**). However the 5’ UTR tree revealed that the isolates were present in two distinct groups, i.e, five porcine BVD isolates were interspersed within BVDV type 1 cattle isolates and among them 4 porcine BVD isolates were close to previously reported Indian cattle isolates of BVDV 1and a single porcine BVD isolate was close to the Turkey isolates of cattle BVDV-1 and three porcine BVD isolates were present as an outlier cluster. The data clearly reveals that the porcine BVD isolates from Meghalaya show closeness to the cattle BVDV isolates of Indian origin.

## 4 DISCUSSION

Since 1960 there were a lots of work done related to pigs infected with BVDV. CSFV antibodies were found in pigs having no clinical signs raised questions and subsequently it was confirmed that the causative agent might be BVDV [15]. After a long waiting BVDV could be succefully isolated from naturally infected pigs in 1973 by Fernelius *et al* but it was already reported back in 1968 by Snowdon and French. Detection of BVDV antibodies were used for classical swine fever quarantine [16]. It was also confirmed that clinical cases showing symptoms of CSF were actually caused by natural BVDV infection [17]. Early reports suggests that BVDV infection caused in pigs due to hog cholera vaccines contaminated with BVDV [18]. The presence of BVDV antibodies in pigs in Austria and Germany has been confirmed at 3–40% and in Holland at 15–20% [19], [20], [21]. Seroprevalence of BVDV in domestic pigs was also recorded in Norway, Denmark, and Ireland respectively at 2.2%, 6.4% and 3.2% [22], [23], [24]. The presence of BVDV antibodies during outbreaks of CSFV in the Netherlands caused havoc interference in the proper serological diagnosis of CSFV [25]. Seroprevalence of BVDV in North American pig were ranging from 2% to 43%, with cattle being demonstrated as the most common source of BVDV infection in pigs [26]. Seroprevalance status of BVDV in pigs in China was considered threatening and it was also confirmed that BVDV1 was predominant strain in China [27].

The effects of intraspecies dynamics as a mediator of spill over is quite intriguing. Feral pigs can potentially become sources for disease transmission because persistent infection can occur in domestic pigs.Several experiments established the presence of BVDV in species other than cattle. Like in mountain goats [28], wild Alpine and Iberian ibex [29]. Reports also shows that domestic goats infected with BVDV shows symptoms like abortion of production of PI kids [30]. Eland or Taurotragus orxy in Zimbabwe near cattle farms were found positive for BVDV antibody [31]. BVDV was also confirmed in Mule deer by IHC and also suspected to be a spillover case [32]. Increasing level of BVDV infection in pigs in the last decade were recorded [26], [33], [34]. BVDV-1 strain ZM- 95 infections in swine population in China were first detected by Wang *et al*. in 1996.

The role of cattle and ruminants in harboring and disseminating BVDV is well documented in India. In the North eastern region of India the practice of composite farming is quite common, it is a matter of practice to rear both the species in close proximity hence facilitating the spread of multi host viruses.

In India, prevalence of BVDV were recorded in cattle, buffaloes, sheep and goats and all the genotypes of BVDV viz, BVDV-1, BVDV-2, and BVDV-3 have been detected [35], [36], [37]. Yaks (Bos grunniens) have been found positive for BVDV in Arunachal Pradesh state [38] and Bos frontalis also known as mithun have been found positive for BVDV antibody from Nagaland, Mizoram and Arunachal Pradesh [39]. There are earlier reports of BVDV in cattle (37.6%), buffaloes (30.76%), sheep (23.4%), and goats (16.9%) in India [40].

In a related study we have screened a total of 916 cattle serum originating in the north east India (especially from nearby places from where pig serum were collected) for the presence of BVDV with Ingezim BVDV compact ELISA for Antibody detection during the period of 2013 to 2015. 113 cattle serum were positive in Ab ELISA and 42 out of 113 cattle serum screened were showing positive result in RT-PCR technique as described by Sandvik *et al* in 1997 [12] (Communicated). In the present study, a total of 10 (out of 206 porcine serum samples) genomic isolates of porcine origin- BVDV was detected by the amplification of partial 5’UTR region of the ORF.

As partial 5’UTR is the mos dominant gene fragment, it has been used widely for phylogenetic analysis of geographically diverse strains of BVDV from field samples [41], [42], [43], [36], [44].

Additionally, E2 have been found useful for more accurate phylogenetic analysis in segregating BVDV into its subgenotypes [41], [42], [44], [45], [6]. Main circulating genotype of BVDV in cattle of India is BVDV-1b [35] while BVDV type 2 occurred sporadically in India [35]. Identification of BVDV-2 in sheep and goats [46], [36] demanded a search in cattle considering migration and trading in ruminants from the porous borders.HoBi like pestivirus or BVDV type 3 also detected from cattle serum in India in 2014 [37].

In phylogenetic analysis it could be seen that on the basis of E-2 sequence alignment all the 8 isolates clustered in one group that was close to BVDV type 1 (Fig. **5**). However the same isolates on the basis of 5’- UTR alignment clustered in two groups *viz*, five isolates with BVDV type 1 and three isolates as a distinct outlier group (Fig. **4**). The E-2 alignment indicated closeness to Cattle BVDV type 1. However, further studies on subtyping the isolates on the basis of full length 5’-UTR are underway. Regarding molecular phylogenetic studies of pestiviruses in the north eastern part of India, several reports have been published stating prevalence of CSFV subgenotype 1.1 and emergence of subgenotype 2.2 in the region [47]. Recently, CSFV of genotype 2.2 from wild hog has also been reported from the region [48]. Overall results in our laboratory showed that the CSFV isolates from India, Nepal and China might have some common ancestor and subgenotype 2.2 is wide spread in the north- eastern region of India.

The occurrence of BVDV in pigs in a single cluster with closeness to BVDV-2 and 1 of cattle on the basis of E-2 sequencing and closeness to BVDV-1 of cattle on the basis of 5’UTR sequencing could indicate emergence and niche adaptation of the virus, as Type 1 BVDV is more genetically variable than type 2 [12]. However if the phylogenetic analysis of 5’-UTR sequences are considered exclusively then the presence of an outlier group closer to CSFV1.1 genogroup could indicate an emergence of a genetically divergent type of BVDV that is establishing itself in the swine population of the north east. This could well be the isolates that would be encountered in the future. As the 5’UTR is relatively stable when compared to E-2; such a change could very well signal an initial adaptation event that could subsequently translate to presumable changes in the E-2 domain after a more prolonged adaptation of BVDV in pigs the north eastern part of India.

## CONCLUSION

The study is the first of its kind regarding identification and preliminary molecular epidemiology of BVDV isolates from porcine in the north eastern part of India, under conditions of integrated and mixed livestock farming. Our study indicates a very minute prevalence of such infections (<5%) from nearly 200 samples analysed over a period of two years. However it can be seen that all the partial E2 gene sequences (8 nos) are close to the BVDV type 1 but forming a distinct cluster. The same isolates show a difference/variation on the basis of 5’ UTR gene sequences (10 nos) and are close to the BVDV type 1. Sentinel surveillance for both BVDV type 1 and 2 isolates need to be established to study the emergence of these isolates in the swine population of the north east. There has to be a heightened surveillance for atypical CSF cases that could indicate emergence of a new strain of BVDV (most likely type 1) that is stabilising itself in the porcine population of the north east.

## Figures and Tables

**Table 1:** List of primers used for 5’ UTR and E2 partial region.

**Name**	**Sequence**	**Position**	**Reference**
PnPestF(P1034)	TAG CCA TGC CCT TAG TAG GAC T	103 - 124	[11]
PnPestR (P365R)	TGT GCC ATG TAC AGC AGA GAT T	386 - 365	
PnCSFF(S174F)	CGT CAG TAG TTC GAC GTG AGC A	174 - 195	
PnCSFR(S285R)	TAT CAG GTC GTA CCC CCA TCA C	306 - 285	
PnBVDVF(B145F)	AAC AGT GGT GAG TTC GTT GGA T	145 - 166	
PnBVDVR(B314R)	CAC CCT ATC AGG CTG TAT TCG T	335 - 314	
E2F(B11)	TAA GAC CA(A/G) ATT GGT GGC CTT ATG AGA C	2247 - 2274	[12]
E2R(B32)	GGG CA(A/T) ACC AT(C/T) TGG AAG GC(C/T) GG	2853 - 2831	

**Table 2:** Result of Taqman qPCR detection kit for BVDV.

**Sample Name**	**Target Name**	**Reporter**	**Status of sample**	**Cт**
S1118	BVDV	FAM	Unknown	20.51847
MzP1	BVDV	FAM	Unknown	20.00011
MzP2	BVDV	FAM	Unknown	21.1311
MzP3	BVDV	FAM	Unknown	22.74387
MzP4	BVDV	FAM	Unknown	27.78656
MzP7	BVDV	FAM	Unknown	26.58849
MzS1	BVDV	FAM	Unknown	18.37358
ML1920	BVDV	FAM	Unknown	18.73578
AR27	BVDV	FAM	Known Negative	Undetermined
AS243	BVDV	FAM	Known Negative	Undetermined
AS342	BVDV	FAM	Known Negative	Undetermined
AR6	BVDV	FAM	Known Negative	Undetermined
ML1915	BVDV	FAM	Unknown	20.18769
ML1879	BVDV	FAM	Unknown	20.13571
Mz470	BVDV	FAM	Known Negative	Undetermined
AR11	BVDV	FAM	Known Negative	Undetermined
ML1910	BVDV	FAM	Known Negative	Undetermined
S444	BVDV	FAM	Known Negative	Undetermined
S473	BVDV	FAM	Known Negative	Undetermined
S468	BVDV	FAM	Known Negative	Undetermined
NTC	BVDV	FAM		Undetermined

The detailed results of Real-Time PCR are shown in Fig. (**3**) and in Table **2**.
